# Virus-Clip: a fast and memory-efficient viral integration site detection tool at single-base resolution with annotation capability

**DOI:** 10.18632/oncotarget.4187

**Published:** 2015-05-19

**Authors:** Daniel WH Ho, Karen MF Sze, Irene OL Ng

**Affiliations:** ^1^ Department of Pathology, The University of Hong Kong, Hong Kong, China; ^2^ State Key Laboratory for Liver Research, The University of Hong Kong, Hong Kong, China

**Keywords:** viral integration, breakpoint detection, viral integration site detection, next-generation sequencing

## Abstract

Viral integration into the human genome upon infection is an important risk factor for various human malignancies. We developed viral integration site detection tool called Virus-Clip, which makes use of information extracted from soft-clipped sequencing reads to identify exact positions of human and virus breakpoints of integration events. With initial read alignment to virus reference genome and streamlined procedures, Virus-Clip delivers a simple, fast and memory-efficient solution to viral integration site detection. Moreover, it can also automatically annotate the integration events with the corresponding affected human genes. Virus-Clip has been verified using whole-transcriptome sequencing data and its detection was validated to have satisfactory sensitivity and specificity. Marked advancement in performance was detected, compared to existing tools. It is applicable to versatile types of data including whole-genome sequencing, whole-transcriptome sequencing, and targeted sequencing. Virus-Clip is available at http://web.hku.hk/~dwhho/Virus-Clip.zip.

## INTRODUCTION

Viral infection is a common risk factor for various human malignancies [1]. Particular viruses e.g. hepatitis B virus (HBV) can integrate into the human genome upon infection and lead to disruption in gene functions that predispose to carcinogenesis. In the past, PCR-based methods were employed to detect viral integration events. As a result of limited sensitivity and resolution, the efficiency of detection was restrained. This major obstacle was solved due to the recent advancement in next-generation sequencing (NGS). Since NGS data is large, manual inspection is impossible. This imposes huge demand on useful tools for the task. Existing tools provide useful resources in identifying viral integration events but there are still limitations remained unsolved. For instance, VirusSeq [2] cannot report the exact human and virus breakpoint positions. Besides, ViralFusionSeq [3] and VirusFinder [4, 5] involve sophisticated installation procedures and long execution time, which hinder their practical use. In addition, not all the existing tools are having annotation function on the affected human genes.

**Figure 1:**
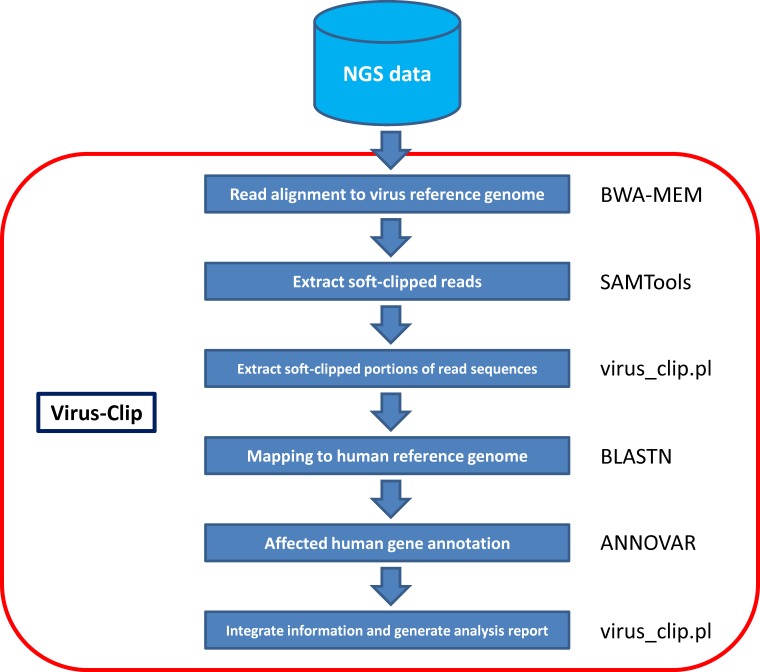
Workflow of Virus-Clip

Here, we present our viral integration detection tool, namely Virus-Clip. Virus-Clip makes use of the virus genome as the primary read alignment target. Then, it extracts soft-clipped reads from the alignment and maps the soft-clipped segments (potentially containing sequences of HBV-integrated human loci) to the human genome. Making use of the mapping information, Virus-Clip can report the human and virus integration breakpoints to single-base resolution. Besides, all the integration sites are automatically annotated with the affected human genes and their corresponding gene regions. With streamlined procedures involving minimal steps and tools, Virus-Clip delivers a simple, fast and memory-efficient solution to viral integration site detection (Figure 1). Execution performance demonstrated a significant advancement, compared to existing tools. Virus-Clip is available at http://web.hku.hk/~dwhho/Virus-Clip.zip.

## RESULTS

To evaluate the performance of Virus-Clip, we applied it to whole-transcriptome sequencing (RNA-seq) data of two human HBV-associated hepatocellular carcinoma (HCC) samples. The RNA-seq data was generated by 101bp paired-end Illumina HiSeq 2000 platform. Viral integration site detection was similarly performed by VirusFinder and ViralFusionSeq with default parameters. VirusSeq was not included in the comparison as it cannot report exact breakpoint positions. Raw execution result data is available as Supplementary Data. Performance comparison was undertaken on the basis of speed, computer resources requirement and viral integration site identification outcome (Table 1).

**Table 1:** Benchmark result for viral integration site detection

Sample	Tool	# of reads (M)	Execution time (min)	# of CPU	Memory used (GB)	# of viral integration events	Key affected human gene
HBV-associated HCC 1	Virus-Clip	63.8	35.9	1	2.1	8	*KMT2B*
	VirusFinder		244.6	8	15.9	1	
	ViralFusionSeq		Execution failure				
HBV-associated HCC 2	Virus-Clip	70.0	36.4	1	2.4	14	*TERT*
	VirusFinder		259.3	8	15.8	3	
	ViralFusionSeq		Execution failure				

Note: Error encountered during ViralFusionSeq execution

Virus-Clip identified 8 and 14 HBV integration sites respectively for the two studied samples while 1 and 3 sites were found by VirusFinder. ViralFusionSeq was failed to execute on our dataset but its execution could finish on its example data, suggesting there was no installation error.

**Figure 2:**
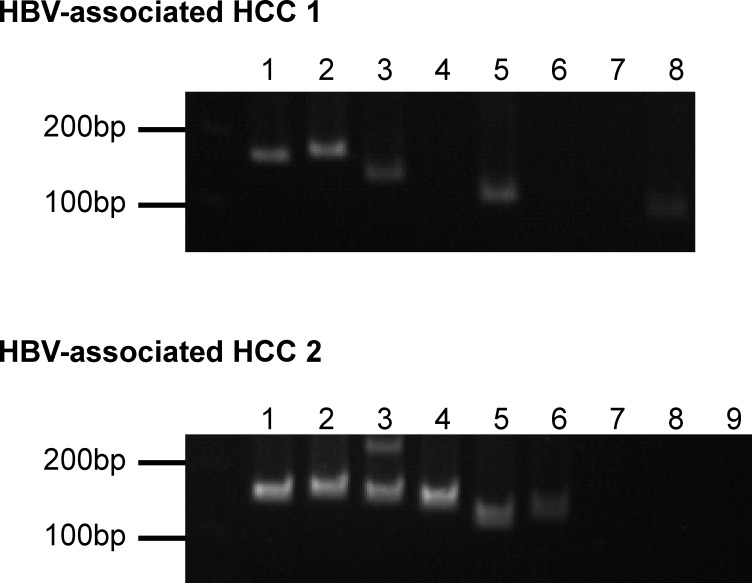
PCR amplification of selected HBV integration events The lanes correspond to the integration events listed in Table 2. Order of integration events is sorted according to supporting read count with the leftmost one supported by the most.

In the context of HBV integration into human genome, locations upstream of *TERT* gene and inside *KMT2B* gene were frequently reported on HBV-associated HCC [6]. These two key HBV integration events were found in the two studied samples respectively and were successfully identified both by Virus-Clip and VirusFinder. Therefore, both tools were able to identify key viral integration events. Nevertheless, the numbers of supporting soft-clipped reads on the *TERT* integration event were 12 and 6, while they were 17 and 8 on the *KMT2B* integration event, for Virus-Clip and VirusFinder respectively. To further evaluate the sensitivity and specificity of the detection by Virus-Clip, we selected 17 HBV integration events supported by at least 1 soft-clipped sequencing read and designed primers that flank the identified HBV integration junctions (Table 2). The validity of the integration events was related to the supporting read count. Most of the events (9 of 10 or 90%) supported by more than 2 soft-clipped sequencing reads were successfully validated while the validated proportion was still pretty high (10 of 14 or 71.4%) when the threshold was set at 2 soft-clipped sequencing reads (Figure 2 and Table 2). Using a stringent threshold of more than 2 soft-clipped sequencing reads, Virus-Clip still reported more HBV integration events than VirusFinder, suggesting a higher sensitivity of the former over the latter. More importantly, the validated proportion was concomitantly high, indicating high specificity or minimal false-positive reports. Based on the empirical data, we recommend 2 soft-clipped sequencing reads as a sensible threshold for preliminary filtering of viral integration events reported by Virus-Clip. Taken together, lines of evidence suggest the superior sensitivity and specificity of Virus-Clip and it allows the potential detection of rarer viral integration events that are supported by fewer sequencing reads. More importantly, in terms of speed, CPU and memory usage, and the total number of viral integration events identified, Virus-Clip outperformed VirusFinder. Hence, Virus-Clip represents a significant improvement on existing viral integration site identification tools.

**Table 2:** Validation experiment on 17 selected HBV integration events

Sample	Lane in Figure 2	Integrated genic region	Affected human gene	Supporting read count	Forward primer	Reverse primer	PCR product size (bp)	Validated
HBV-associated HCC 1	1	intronic	*KMT2B*	17	GGAGGAGTTGGGGGAGGAG	CTGGAAAGTGTCCAAGGAGG	162	Yes
	2	exonic	*KMT2B*	7	CTCAAGAGAGCCAAAGTGCAGC	ACACAGAATAGCTTGCCTGAG	170	Yes
	3	splicing	*KMT2B*	6	AATTTGTCCTGGTTATCGCTGG	CTCCGGCCACCTCCTCCATCTGC	141	Yes
	4	UTR3	*TJAP1*	5	GCAACCTGCTCAACTAGGGCCCCTGCTG	GATTACATATCCCATGAAGTTAAGG	147	No
	5	intronic	*KMT2B*	2	AGCAGAAGGTGGCAGCTTCCATG	CGGGTCAATGTCCATGCCCCAAAGC	116	Yes
	6	UTR5	*ZNF792*	1	TCTCGCAGCGCCGCCGCTGCCATC	AGACGGGGAGTCCGCGTAAAGAGAG	101	No
	7	intergenic	-	1	CTTTAATTAGTATCTTCTAC	GGCCATTGATCCGTGTTGG	101	No
	8	exonic	*KMT2B*	1	TGGACTTTCAGCAATGTCAACG	GATCTGCTTGACATCCCCGGCCAC	101	No
HBV-associated HCC 2	1	upstream	*TERT*	37	ATCCCAGTAGAGTAGGAG	CAAATACTCAAGAACAGTTTC	148	Yes
	2	upstream	*TERT*	15	GGCGAGAAACTTCTGGGTCTC	GCATTTGGTGGTCTGTAAGC	154	Yes
	3	exonic	*TERT*	12	GCTGGATGGGTCGGCGGCG	GCAGGAACTTGGCCAGGATC	150	Yes
	4	intergenic	-	9	ACCAACATTTGAACAGTCACC	TACGGGTCAATGTCCATGCC	134	Yes
	5	exonic	*TERT*	7	GCGGCGTTTTATCATCTTCC	GCACAGCCTCTGCAGCACTC	112	Yes
	6	intergenic	-	4	GAGTTGGGGGAGGAGATTAG	GTTTCTGAGCTCTGTCAAAACGG	154	Yes
	7	UTR5	*NUMB*	2	GTTTTATCATCTTCCTCTTCATCCTG	CTTGAATTGTAACAGTGGCTGC	132	No
	8	intergenic	-	2	GGAGATTAGGTTAAAGGTC	GCCAAAGTTAAGGACACTCTTGTGAC	113	No
	9	exonic	*HP*	2	CTTTGGAAGAGAAACTGTTCTTGAG	GGACTGTGCTGCCTTCATAATGCC	109	No

It summarizes the details of the events and corresponds to the PCR amplification in Figure 2. Integration events are sorted according to supporting read count, with those successfully amplified by PCR and confirmed by Sanger sequencing remarked as validated in the rightmost column.

## DISCUSSION

The availability of NGS technology opens up the possibility of systematic and unbiased examination of viral integration event. Although existing analysis tools allow the screening of NGS data at great resolution, the huge data size imposes severe demand on the computational resources and requires long execution time. These major obstacles make some of the existing tools not suitable in analyzing whole-genome sequencing (WGS) data of extremely large size. With the strategy of initial read alignment to virus reference genome instead of human reference genome and streamlined procedures involving only a few essential tools, these issues lead to the superior performance of Virus-Clip. Due to the relatively small size of virus genome, the alignment to it is significantly more efficient. Moreover, Virus-Clip makes use of BWA-MEM for initial alignment to virus genome, SAMTools for soft-clipped reads extraction, BLASTN for local alignment of human chimeric fragment to human genome, and ANNOVAR for annotation. Such minimal combination of tools and workflow allows streamlined procedures. Virus-Clip substantially shortened the process and time in analyzing viral integration event. It also requires significantly fewer computational resources. The installation of Virus-Clip is also simplified, as a result of the simple overall workflow. Furthermore, the automatic annotation capability of the integration sites can facilitate the practical use of the obtained viral integration information. Therefore, to our best knowledge, Virus-Clip contributes a major advancement in viral integration site identification. It provides a simple, fast and memory-efficient solution to identify viral integration event at single-base resolution that requires minimal computer resources and applicable to versatile types of NGS data including WGS, RNA-seq and targeted sequencing. Apart from the RNA-seq data mentioned above, we have also applied Virus-Clip on targeted DNA sequencing data. Similarly satisfactory performance could be obtained (data not shown). One limitation of Virus-Clip is that it requires the provision of virus reference genome as input and hence it is not applicable to data without virus reference genome available (which is unlikely in most circumstances).

## MATERIALS AND METHODS

### Implementation of Virus-Clip

Virus-Clip is implemented in shell script that executes third-party tools and our own Perl program (Figure 1). The viral integration site identification relies on soft-clipped sequencing reads that represent chimeric fusion of human and virus genomic sequences. It can accept both single-end and paired-end sequencing reads in FASTQ format.

Virus-Clip consists of a shell script (Virus_Clip.sh) that executes third-party tools and our own Perl program (virus_clip.pl). The actual procedure involves 3 major steps. First, it maps sequencing reads to virus reference genome by Burrows-Wheeler Aligner (BWA-MEM) [7], which is capable of soft-clipped alignment. As the size of virus reference genome is far smaller than the human reference genome, this step can effectively narrow down the search space in the initial alignment and lead to significantly shortened execution time and reduced computational resources when compared with initial alignment to human reference genome.

Then, with the use of SAMTools [8], it examines the alignment of Sequence Alignment/Map (SAM) format and extracts soft-clipped reads from it, through utilizing the CIGAR flag. Other information such as the mapping position and aligned sequence are obtained from the SAM columns. Information is stored as a temporary file.

Finally, Perl program virus_clip.pl reads the temporary file and obtains the soft-clipped portions of the reads (potentially including the flanking human genomic sequence that the virus integrated at). It subsequently maps them to the human reference genome by the BLASTN stand-alone version (available at ftp://ftp.ncbi.nlm.nih.gov/blast/executables/blast+/LATEST/) with default parameters. Top match (if any) is reported as the virus integrated location. Using ANNOVAR [9], annotation information on the affected human gene region and the affected human gene were obtained. In the result file (virus_clip.out), information on the human and virus integration loci, the corresponding flanking sequences, the number of supporting soft-clipped reads, and the affected human genes and their regions are reported.

### Validation experiment on HBV integration events detected by Virus-Clip

We selected 17 HBV integration events supported by at least 1 soft-clipped sequencing read and designed primers that flank the identified HBV integration junctions (Table 2). To confirm the validity of the PCR amplicons, they were subjected to Sanger sequencing and confirmed to match with the detected chimeric fragment sequences.

## SUPPLEMENTARY DATA








